# Prognostic significance of long noncoding RNA HOTAIR in hepatocellular carcinoma: A protocol for systematic review and meta-analysis

**DOI:** 10.1097/MD.0000000000029406

**Published:** 2022-07-29

**Authors:** Lei Feng, Wenqing Liu, Yunhuo Lv, Baojun Qiao

**Affiliations:** a Peking University International Hospital, International Department, Beijing 102206, China; b China Rehabilitation Research Center, Pulmonary and Critical Care Medicine, Beijing 100068, China; c Department of Oncology, Shangrao Municipal Hospital, Shangrao, China; d Department of Gastroenterology, Baima Outpatient Department, Jingdong Medical District, PLA General Hospital, Beijing, China.

**Keywords:** long noncoding RNA, HOTAIR, hepatocellular carcinoma, prognosis, meta-analysis

## Abstract

**Background:**

Homeobox transcript antisense intergenic RNA (HOTAIR), a long noncoding RNA, has been reported to associate with the prognosis of patients with hepatocellular carcinoma (HCC) in several studies, however, the definite conclusion has not been obtained for conflicting results across different studies. The aim of this study is to determine the association of HOTAIR expression with overall survival, progression-free survival, and clinical features in HCC.

**Methods:**

PubMed, Cochrane Library, and Embase will be comprehensively searched to seek the relevant studies. The studies meeting the inclusion criteria will be included into this systematic review and meta-analysis. A combination of hazard ratio and 95% confidence interval is used to estimate the impact of HOTAIR expression on the overall survival and progression-free survival in HCC. The relationship between HOTAIR expression and clinical features of HCC is evaluated using the odds ratio and 95% confidence interval. The study quality is evaluated with the “risk of bias assessment” tool in Cochrane System Assessment Manual or Newcastle-Ottawa Scale. The subgroup analysis, publication bias, and sensitivity analysis are performed.

**Results:**

This study provides a strict and classic protocol for systematic review and meta-analysis to determine the prognostic significance of HOTAIR expression in HCC. The findings of this systematic review and meta-analysis may provide a novel diagnostic indicator and potential therapeutic target of HCC.

**Ethics and dissemination:**

This study is only a protocol for systematic review and meta-analysis, and all data used in this study is acquired through published studies. Therefore, the ethical review is not needed for this study.

**Registration number:**

INPLASY202230050.

## 1. Introduction

Primary liver cancer has become one of the leading causes of death from cancer worldwide, and hepatocellular carcinoma (HCC) accounts for the most cases of primary liver cancer.^[1]^ Despite the great advancement in the diagnosis and treatment, the prognosis of patients with HCC remains disappointing, with the overall 5-year survival rate less than 12% reported.^[2]^ To improve the prognosis of HCC, researchers began to seek the appropriate biomarkers to serve as the diagnostic indicators and potential targets for the management of HCC.^[3–6]^

Long noncoding RNA (lncRNA) refers to a class of regulator RNA longer than 200 nucleotides in length without the capacity of encoding proteins.^[7]^ It has been proved that lncRNAs played important roles in the tumor genesis, invasion, and metastasis of human cancers,^[8–10]^ including HCC.^[11–13]^ Several lncRNAs have been identified to serve as the prognostic indicators for HCC, such as TUG1,^[12]^ LINC01234,^[11]^ and SNHG5.^[14]^ Recently, homeobox transcript antisense intergenic RNA (HOTAIR), a lncRNA, has been verified to be associated with the prognosis of HCC.^[13,15–22]^ Han et al. study showed high HOTAIR expression was an independent prognostic factor of overall survival (OS) (*P* < .01) and progression-free survival (PFS) (*P* < .01) in patients with advanced HCC treated by sunitinib.^[18]^ Similarly, in Ishibashi et al. study, the authors observed that HCC patients in high HOTAIR expression had a larger tumor size compared with those in low HOTAIR expression (*P* < .01).^[19]^ In a like manner, Zhong et al study also reported that high HOTAIR expression was significantly associated with advanced clinical stage (*P* < .01) and earlier distant metastasis (*P* = .02) of HCC, but no significant relationship between HOTAIR expression and tumor size was observed (p = 0.94).^[22]^ In Gao et al. study, the authors reported that high HOTAIR was associated with worse tumor differentiation (*P* < .01), earlier metastasis (*P* < .01), and earlier recurrence (*P* < .01) in HCC, but there was no obvious association of HOTAIR expression with clinical stage (*P* = .09) or tumor size (*P* = .51).^[17]^ Therefore, although the previous studies have presented the potential role of HOTAIR expression in the prognosis of HCC, the conflicting results remained in some clinical features, such as clinical stage and tumor size. Moreover, most of existing evidences were published with a retrospective design in a small population, which inevitably affected the reliability of results.^[17–20]^ Therefore, a systematic review and meta-analysis is urgently needed to determine the prognostic significance of HOTAIR expression in HCC.

This systematic review and meta-analysis is conducted to answer the following questions:

1. Is high HOTAIR expression associated worse OS and PFS in HCC?
2. Is high HOTAIR expression associated with worse clinical features (e.g., more advanced clinical stage) in HCC?

## 2. Methods

The approval of ethics committee and informed consent are not needed for the reason that this study is only a protocol without patient recruitment and personal information collection. This systematic review and meta-analysis is conducted according to the Preferred Reporting Items for Systematic Reviews and Meta-Analysis Protocols.^[23]^ This study has been registered in the International Platform of Registered Systematic Review and Meta-analysis Protocols (https://inplasy.com/) (INPLASY202230050).

### 2.1. Eligibility criteria

The study selection will be conducted according to the following eligibility criteria by 2 researchers independently, and the dispute will be solved by the discussion with the third researcher.

1. The inclusion criteria are as follows:
  (a) Participants: Patients with HCC older than 18 years;
  (b) Intervention: Patients in the high HOTAIR expression;
  (c) Comparison: Patients in the low HOTAIR expression;
  (d) Outcomes: OS, PFS, and clinical features, including age, gender, tumor size, tumor number, histopathologic grading, α-fetoprotein level, cirrhosis, portal invasion, lymph node metastasis, distant metastasis, and clinical stage.
  (e) Study design: Prospective or retrospective studies.
2. The exclusion criteria include case reports, reviews, animal or cell experiments, incomplete data, and duplications.

### 2.2. Literature search and selection

PubMed, Cochrane Library, and Embase will be comprehensively searched to seek the relevant studies using the following key words: “long noncoding RNA,” “lncRNA,” “homeobox transcript antisense intergenic RNA,” “HOTAIR,” “liver cancer,” “hepatocellular carcinoma,” “HCC,” “prognosis,” and “survival.” There is no restriction on the language. The literature search plan in the PubMed database is showed in Table 1, and the search strategy will be modified in the other 2 databases. The references of retrieved studies will also be checked to avoid missing relevant studies.

**Table 1:** Details of literature search in PubMed.

Search number	Query	Results
#1	Long noncoding RNA	
#2	lncRNA	
#3	#1 OR #2	
#4	Homeobox transcript antisense intergenic RNA	
#5	HOTAIR	
#6	#4 OR #5	
#7	Hepatocellular carcinoma	
#8	HCC	
#9	#7 OR #8	
#10	Prognosis	
#11	Survival	
#12	#10 OR #11	
#13	#3 AND #6 AND #9 AND #12	

**Figure 1.**
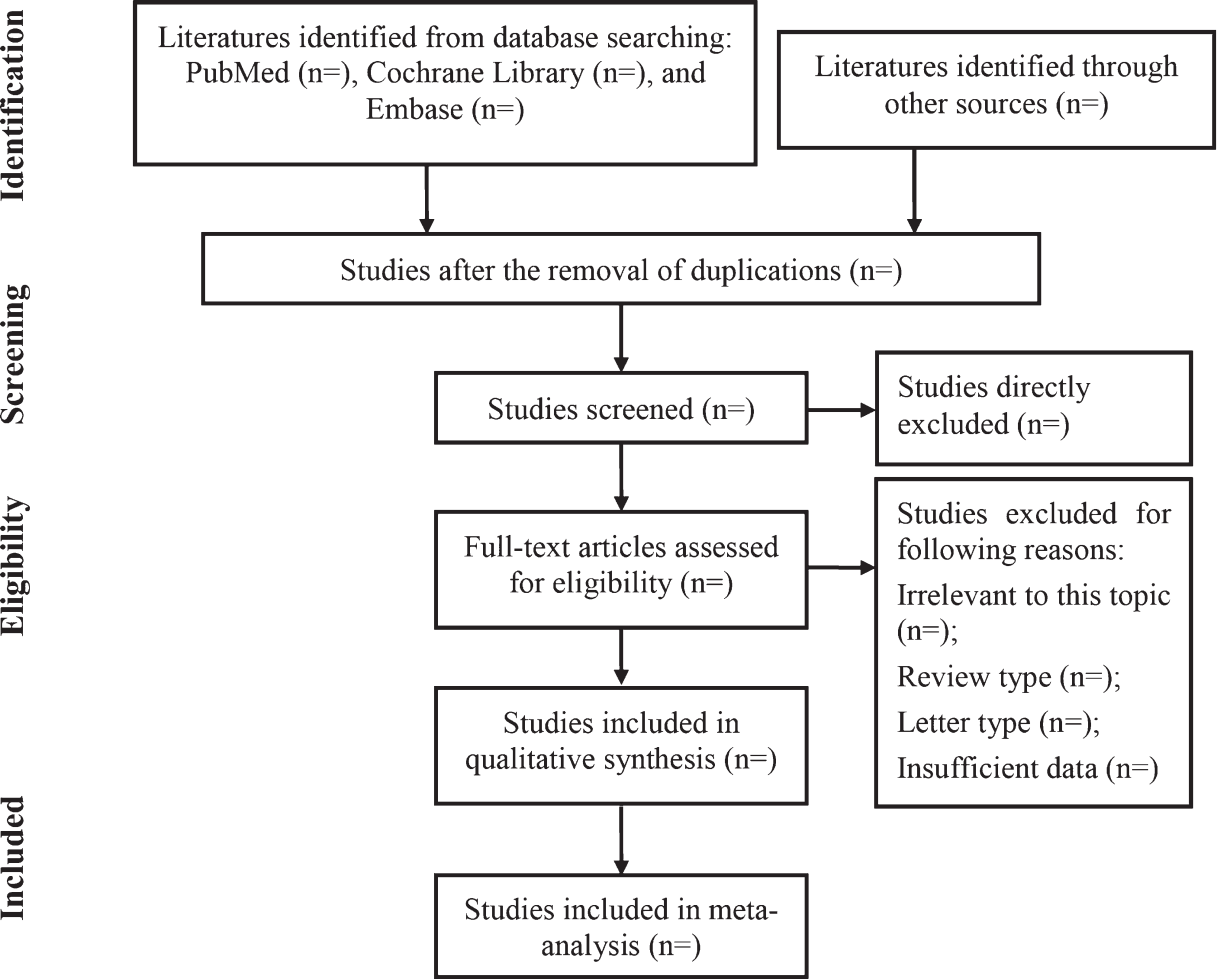
The flow chart for literature search and study selection.

Then, the retrieved studies will be selected using the abovementioned eligibility criteria, and the detailed flow chart is showed in Figure 1.

### 2.3. Data extraction

The following information will be extracted from each included study independently by 2 researchers.

1. Information for published studies, including the author’s name, publication year, title of the study, publication journal, and sample size.
2. Prognostic indicators, including OS and PFS;
3. Clinical features, including age, gender, country, tumor size, tumor number, histopathologic grading, α-fetoprotein level, cirrhosis, portal invasion, lymph node metastasis, distant metastasis, and clinical stage.
4. Information used for assessing the quality of included studies.

### 2.4. Quality assessment

Two tools are used to evaluate the quality of included studies. For the randomized controlled trial (RCT), the “risk of bias assessment” tool in Cochrane System Assessment Manual will be used to evaluate the risk of bias of included studies.^[24]^ Using this standard, 1 RCT study will be divided into 3 classes: high, unclear, or low risk of bias. For non-RCT study, the quality of included study will be assessed with the Newcastle-Ottawa Scale score, and the study with Newcastle-Ottawa Scale score ≥7 is considered to have the high quality.^[25]^ The quality assessment of included studies is conducted by 2 researchers independently, and any disagreement will be solved by the discussion with the third researchers.

### 2.5. Statistical analysis

All analyses in this study will be performed with Review Manager (RevMan) 5.3 and Stata 12.0 (Stata Corp, College Station, TX). A combination of hazard ratio (HR) and 95% confidence interval (CI) is used to estimate the impact of HOTAIR expression on the OS and PFS in HCC. The relationships between HOTAIR expression and clinical features of HCC are evaluated using the odds ratio (OR) and 95% CI. If one study only reported the Kaplan–Meier curve, the HR and corresponding 95% CI will be calculated using the methods provided by Tierney et al.^[26]^ All results will be showed in the form of forest plots. A two-side p-value less than 0.05 indicates there is a statistically significant result.

### 2.6. Assessment of heterogeneity

A test of heterogeneity of combined HR or OR was conducted using the Cochran Q test and Higgins *I*^2^ statistic. A *P* value for heterogeneity less than .10 or *I*^2^ larger than 50% indicates there is an obvious heterogeneity among included studies, as a result, a random-effect model should be used. On the contrary, a *P* value for heterogeneity larger than .10 or *I*^2^ less than 50% indicates there is no obvious heterogeneity among included studies, and a fixed-effect model should be used.

### 2.7. Subgroup analysis

To comprehensively evaluate the relationship between HOTAIR expression and OS, the subgroup analyses of OS will be performed using different grouping standards, such as country, sample size, treatment therapy, and quality of included studies.

### 2.8. Publication bias

The funnel plot is generated to evaluate the publication bias of included studies, and is further quantitatively analyzed using the Egger test and Begger test.

### 2.9. Sensitivity analysis

The sensitivity analysis is performed to determine that whether one of the included studies has the decisive effect on the pooled results. The sensitivity analysis is conducted using the Stata 12.0 by removal of one study one time to observe the change of pool results.

## 3. Discussion

HCC has become one of the leading causes of cancer patients for the poor prognosis, especially patients at advanced clinical stage.^[1,2]^ A growing number of researchers begin to seek appropriate biomarkers to serve as diagnostic indicators and potential therapeutic targets for HCC with the hope of improving the prognosis of patients with HCC.^[3–6]^ Recently, increasing evidences showed that HOTAIR, a lncRNA, was significantly associated with the tumor genesis, progression, and metastasis of HCC via different underlying mechanisms. Liu et al study reported that HOTAIR might promote the HCC progression by regulating the miR-526b-3p/DHX33 axis.^[20]^ Similarly, Tang et al. study presented that HOTAIR contributed to the sorafenib resistance via suppressing the miR-217 in HCC.^[21]^ In Duan et al study, the authors reported that HOTAIR might lead to the Taxol-resistance of HCC cells by activating the AKT phosphorylation and down-regulating the miR-34a.^[16]^ Moreover, Cheng et al study showed that HOTAIR could epigenetically suppress the miR-122 expression in HCC via DNA methylation.^[15]^ Besides, Topel et al found that the overexpression of HOTAIR could induce the downregulation of c-Met signaling and promote the hybrid epithelial/mesenchymal phenotype in HCC cells.^[13]^ Therefore, plenty of studies have indicated that HOTAIR might promote the progression of HCC via different biological mechanisms. Based on these findings, researchers begin to explore the prognostic significance of HOTAIR in HCC, however, contradictory results were reported from different studies.^[17–19,22]^ Therefore, we conduct this systematic review and meta-analysis by integrating the existing evidences to further determine the prognostic significance of HOTAIR expression in HCC. To our knowledge, this study is the first systematic review and meta-analysis to explore the role of HOTAIR in the prognosis of patients with HCC. We hope our promising findings in this study can provide reliable evidences on the prognostic significance of HOTAIR expression in HCC.

## Acknowledgments

We would like to thank the researchers and study participants for their contributions.

## Author contributions

Conceptualization: Baojun Qiao.

Data curation: Lei Feng, Yunhuo Lv.

Formal analysis: Lei Feng.

Investigation: Lei Feng, Wenqing Liu, Yunhuo Lv.

Methodology: Lei Feng, Wenqing Liu.

Resources: Lei Feng, Wenqing Liu, Yunhuo Lv.

Software: Wenqing Liu.

Supervision: Baojun Qiao.

Validation: Yunhuo Lv.

Visualization: Yunhuo Lv.

Writing – original draft: Lei Feng, Wenqing Liu, Yunhuo Lv.

Writing – review & editing: Baojun Qiao, Lei Feng.
